# Function, strength, and muscle activation of the shoulder complex in Crossfit practitioners with and without pain: a cross-sectional observational study

**DOI:** 10.1186/s13018-022-02915-x

**Published:** 2022-01-15

**Authors:** Elisa Raulino Silva, Nicola Maffulli, Filippo Migliorini, Gilmar Moraes Santos, Fábio Sprada de Menezes, Rodrigo Okubo

**Affiliations:** 1 Departamento de Fisioterapia, Universidade do Estado de Santa Catarina, Florianópolis, SC Brasil; 2 Department of Musculoskeletal Disorders, Faculty of Medicine, Surgery and Dentistry, Salerno, Italy; 3 Centre for Sports and Exercise Medicine at Queen, Mary University of London, London, UK; 4 Institute of Science and Technology in Medicine, Keele University School of Medicine, Stoke-on-Trent, UK; 5 Department of Orthopaedic, Trauma, and Reconstructive Surgery, RWTH Aachen University Hospital, Pauwelsstraße 30, 52074 Aachen, Germany; 6 Programa de Pós-Graduação em Fisioterapia, Universidade do Estado de Santa Catarina, Florianópolis, SC Brasil

**Keywords:** Crossfit, Shoulder, Pain, Mobility, Muscle activation, Shoulder function

## Abstract

**Background:**

The shoulder joint is the most commonly injured joint in CrossFit practitioners, because of the high intensity and loads associated with this sport. Despite the large number of clinical cases, there is a shortage of studies that investigate influence of biomechanical aspects of upper limbs' injuries on CrossFit practitioners. This study hypothesized that there would be a difference in function, strength, and muscle activation between Crossfit practitioners with and without shoulder pain.

**Methods:**

We divided 79 Crossfit practitioners into two groups according to whether they reported pain (*n* = 29) or no pain (*n* = 50) in the shoulder during Crossfit training. Muscle function, strength, and activation were assessed using the Disability Arm, Shoulder and Hand function questionnaire, Upper Quarter Y Balance Test and Closed Kinetic Chain Upper Extremity Stability Test shoulder tests, isometric muscle strength assessment by manual dynamometry and muscle activation by surface electromyography and pain report.

**Results:**

The function based on questionnaire was associated with pain (*p* = 0.004). We observed a statistically significant difference between the two groups only in the surface electromyography activity of the lower trapezius, and in the variables of shoulder pain and function (*p* = 0.038).

**Conclusion:**

Crossfit practitioners with shoulder pain occurring during training showed good function and stability of the shoulder joint, but there was a reduction in the activation of stabilizing muscles, especially the lower trapezius.

*Trial registration* Registro Brasileiro de Ensaios Clinico (Brasilian National Registry) with the ID: RBR-2gycyv.

## Introduction

Several biomechanical factors that directly influence joint mechanics and stability are associated with an increased rate of injury and pain in the shoulder [1–7]. Several studies investigate the biomechanics and possible relationships with injury rates in sports such as tennis, volleyball, and swimming that involve great range of motion, speed, and deceleration of the shoulder [8–11]. Crossfit is based on high intensity high load workouts; it requires mobility and stability of the whole body to perform the exercises. The shoulder is most commonly injured in Crossfit, but few studies analyse the biomechanical characteristics of the shoulder in this sport [12–17]. Some validated, reliable, and available tools to evaluate function, such as the Closed Kinetic Chain Upper Extremity Stability Test (CKCUEST) and the Upper Quarter Y Balance Test (UQYBT), allow to analyse the mobility and stability of the trunk and shoulder complex [18–23]. Surface electromyography (sEMG) and manual dynamometry (MD) enable to assess muscle activity and strength [2, 24–26]. All these functional assessment tools have not been used to assess Crossfit practitioners. This study compared shoulder function, strength, and scapulothoracic muscle activation in Crossfit practitioners who reported shoulder pain and in those who did not report any shoulder pain during training.

## Material and methods

### Design

We undertook an observational, descriptive, cross-sectional comparative study, approved by our local Research Ethics Committee (ID 2799959/2018). The evaluations were carried out in five Crossfit training centres, with the authorization of those responsible. The protocol for the proposed study was registered into the Registro Brasileiro de Ensaios Clinico (Brasilian National Registry) with the ID: RBR-2gycyv. The present study followed the Consolidated Standards of Reporting Trials: the CONSORT statement [27].

### Participants

The participants were recruited through social networks, e-mails, and posters. Only those who agreed and signed the written informed consent form participated in the investigation. The sample was not probabilistic and had intentional character. The inclusion criteria were: (1) Crossfit practitioners of both sexes, (2) aged between 18 and 45 years, (3) who had been practicing the sport for at least 6 months, (4) with or without shoulder pain during training. The exclusion criteria were: (1) previous upper limb surgery, (2) neurological conditions, (3) history of fracture/dislocation in the upper limb in the last year or (4) who practised other sports involving a wide range of shoulder motion.

### Procedures

Participants were informed verbally and in writing of the objectives of the study and data collection procedures. After signing the informed consent form, a demographics questionnaire was administered to collect demographic data and information on training volume and the presence of shoulder pain during Crossfit training. Then, the participants answered a second questionnaire, DASH, and the visual analogue pain scale (VAS) that they could feel in the moment of training (> 0 was considered for pain group). Functional (CKCUEST and UQYBT), strength (MD), and muscle activity (sEMG) tests were performed in a random order. The researcher verbally guided and demonstrated the tests, answering possible questions from the participant regarding the execution of the test themselves. The subjects could interrupt the tests at any time if they experienced pain.

### Disability arm, shoulder and hand questionnaire (DASH)

The DASH questionnaire is used to assess upper limb function in heterogeneous populations, with different levels of dysfunctions and symptoms [28–30]. It is composed of a module of 30 questions on the function of the upper limb and two optional modules, composed of 4 questions each, which address the impact of upper limb symptoms on the ability to play musical instruments / play sports and on the performance of the profession. Each question has 5 answer options considering the severity and function of the limb in the activities of the last week [28]. The score ranges from 0 to 100: the higher the score, the greater the disability. The score of the main module is calculated with the following equation: [(sum of the values of the first 30 questions − 30)/1,2]. Optional modules are calculated separately as follows: [(sum of values − 4)/0.16] [28].

### Upper quarter Y balance test (UQYBT)

A mark is made on the floor, with adhesive tapes, in the shape of a Y, which must contain two angles of 135° and one of 90°, where the participant adopts an initial plank position, with feet shoulder-width apart, hand support in the centre of the marking. With the other hand, the subject is asked to achieve the greatest possible range in 3 different directions: medial, superolateral, and inferolateral. The supporting hand must support the weight of the body, while the opposite hand reaches out. The supporting hand names the movement [10, 31, 32]. Two familiarization tests and 3 valid tests were performed for each direction, with 30 s of rest between each attempt [8, 32]. The score is represented as a percentage of the limb length (% LL), and the reach distance was calculated as follows for each direction: [(average of 3 attempts/limb length) × 100]. The composite score is calculated as follows: [(sum of the means of the 3 directions/3 × length of the limb) × 100]. Limb length refers to the distance from the T1 spinous process to the end of the distal phalanx of the middle finger, and the measurement is performed with the shoulders abducted to 90° and elbows extended [10].

### Closed kinetic chain upper extremity stability (CKCUEST)

The participant starts in a plank position, with the feet shoulder-width apart and the hands resting on the floor on two parallel markings, separated at a distance according to the participant's interacromial length [33]. The athlete is instructed to perform the greatest number of touches from one hand to the other, alternately, in 15 s [20, 22]. The hands must return to the top of the markings, and the participant must maintain the plank position throughout the test. Two familiarization tests and 3 valid attempts were carried out. The evaluator counts the number of touches of the hands, and the score will be based on the average of the three attempts [20, 22, 33].

### Manual dynamometry (MD)

A handheld Lafayette® dynamometer (Lafayette Instruments, Model 01165, Indiana, USA) was used to evaluate the peak shoulder muscles isometric strength [34, 35]. The evaluation was performed with the participant supine and the dynamometer positioned 2 cm below the styloid process for all tests [34, 35].

To evaluate external rotation (ER) and internal rotation (IR) strength, the participant's shoulder was positioned at 90° of abduction and 90° of elbow flexion. To evaluate abduction (ABDU) and adduction (ADDU) strength, the shoulder was positioned at 90° of shoulder abduction, with the elbow in full extension [34, 36, 37]. Two familiarizations and 3 valid attempts were made for each test, performing a maximum isometric contraction for 5 s. The peak force in Newton (N) was recorded, and the mean was calculated [36–39].

### Surface electromyography (sEMG)

The upper trapezius (UT), lower trapezius (LT), and pectoralis major (PM) muscles were evaluated bilaterally [2, 40, 41]. The skin was cleaned with hydrophilic cotton soaked in 70% alcohol before applying the electrodes. For electromyographic signal acquisition and processing, the TeleMyo DTS Desk Receiver® electromyograph (Noraxon U.S.A. Inc., Scottsdale, USA) was used with a digital analogue converter with 16-bit resolution and common mode rejection ratio (RRMC) > 100 dB. The signals were captured at a sampling frequency of 2000 Hz and stored by the MR 3.2 software (Noraxon U.S.A. Inc., Scottsdale, USA).

The maximum voluntary contraction (MVC) of these muscles was recorded against manual resistance applied by the evaluator, for 5 s. For UT, MVC was performed in shoulder abduction up to 60° with the patient seated, the LT with the arm abducted at 90° against resistance in abduction, and PM was evaluated with the patient seated, with shoulder flexion at 90°, against resistance in horizontal abduction [34, 41]. The athletes then performed 3 repetitions of the weightlifting movement (shoulder press) with a 15 kg bar. The data were recorded and analysed at a frequency of 10–500 Hz. A curve smoother, based on RMS, with 100 ms windowing was applied and normalized with the peak of the maximum voluntary contraction recorded. Thereafter, the amounts were represented as a percentage of the MVC (% MVC) [24, 42].

### Data analysis

All the statistical analyses were performed using the SPSS program (SPSS for Windows—version 20.0—SPSS inc.). The Shapiro–Wilk test was performed to verify data distribution. To compare sample characterization data, functional and muscle tests, the *t* test for independent samples was used. To compare the training volume, EVA and DASH between the groups, the Mann–Whitney test was performed. For comparison of categorical variables, the nonparametric Chi-square test of Independence was used, but if the expected frequency of the data is less than 5, the Monte Carlo test was used to assess its likely associations with participants without pain and in pain. The association between independent variables (strength and muscle activity) and dependent variables (clinical tests) was performed using simple linear regression, with the regression coefficient (β) being considered for non-standard continuous measures. The linear regression test was performed separately for the PG sample and for the NPG sample in order to verify different strategies adopted between the groups. Significance was set at *p* < 0.05 for all statistical tests.

## Results

### Recruitment process

**Fig. 1:**
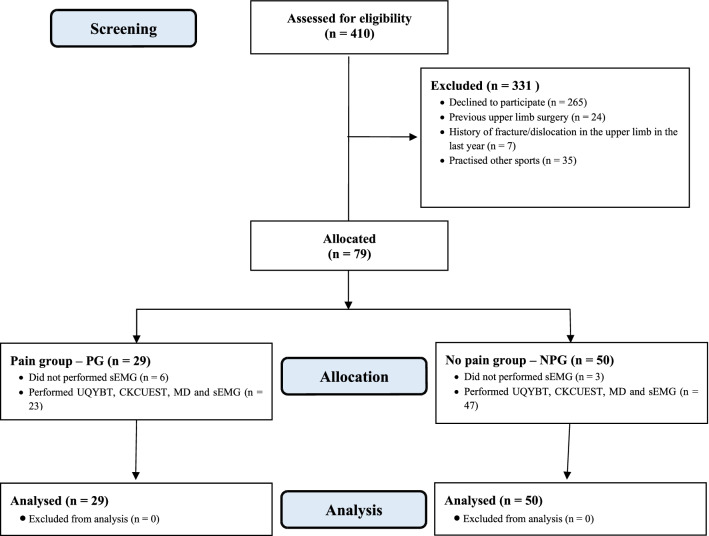
Flow chart of the recruitment process. Patient demographic. The PG and NPG groups did not show significant statistical differences in age, height, weight, time of practice and weekly training (*p* > 0.05) The characteristics of the two groups are shown in Table 1

A total of 410 Crossfit practitioners from 5 selected training centres were initially tested for eligibility. Of them, 265 declined to participate, and 66 were not eligible: previous upper limb surgery (*n* = 24), history of fracture/dislocation in the upper limb in the last year (*n* = 7), practised other sports (*n* = 35). Therefore, 79 participants were included in this study. The Crossfit practitioners included in the study were divided into two groups: group with related shoulder pain (PG *n* = 29) and group without shoulder pain (NPG *n* = 50) during training. During data collection, there were some technical problems involving the sEMG software, and therefore, 9 participants (6 of PG and 3 of NPG) were not evaluated with this instrument. The diagram of the recruitment process is shown in Fig. 1.

### Patient demographic

The PG and NPG groups did not show significant statistical differences in age, height, weight, time of practice and weekly training (*p* > 0.05) The characteristics of the two groups are shown in Table 1.

**Table 1:** Patient demographic

Variable	PG (*n* = 29)	NPG (*n* = 50)	*p*
Sex (male)	58.6% (17 of 29)	64% (32 of 50)	n.s.
Dominance (right)	89.6% (26 of 29)	92% (46 of 50)	n.s.
Pain side (right)	65.5% (19 of 29)	–	n.s.
Age	30.5 ± 5.1	29.9 ± 6.3	n.s.
Height (cm)	171.2 ± 10.1	170 ± 8.3	n.s.
Weight (kg)	74.2 ± 13.1	74.2 ± 10.8	n.s.
Practice time (months)	19.0 [26.0]	24.0 [20.0]	n.s.
Week training (h)	6.0 [4.0]	5.5 [3.0]	n.s.

Age, height and weight values were presented as mean and standard deviation; practice time and week training values were presented as median and interquartile range (cm: centimetres; kg: kilogram; n.s.: not significant)

The PG and NPG values for the variable DASH and CKCUEST are shown in Table 2. Comparison between the groups showed a statistically significant difference in DASH (*p* < 0.05). There was no statistically significant difference for CKCUEST between PG and NPG Crossfit practitioners.

**Table 2:** Comparison of the variable DASH and CKCUEST of PG and NPG

Variable	PG (*n* = 29)	95%CI	NPG (*n* = 50)	95%CI	*p*
*DASH*					
Module	10.8 [13.7]	9.0 to 17.9	2.0 [4.3]	2.3; 5.9	**0.000**
Optional 1	18.7 [31.2]	16.1 to 32.1	0.0 [14.0]	4.7; 14.7	**0.001**
Optional 2	0.0 [25.0]	6.0 to 18.9	0.0 [0.0]	0.1; 6.6	**0.006**
CKCUEST (touches)	25.0 ± 4.6	23.2 to 26.8	24.1 ± 4.9	22.7; 25.5	0.437

No significant difference were found (*p* > 0.1)

DASH values (module, optional 1, optional 2) were presented as median and interquartile range; CKCUEST values were presented as mean and standard deviation

### Intragroup comparison

For the PG group, the shoulder with pain and the shoulder without pain from the same individual were compared. There was no statistically significant difference for UQYBT, MD or sEMG between the side with pain and the side with no pain of the PG. For the NPG group, the dominant upper limb was compared with the non-dominant upper limb of the same individual. There was no statistically significant difference for UQYBT, MD or sEMG between the comparison of the dominant and non-dominant side of the NPG (Table 3).

**Table 3:** Comparison of the variables UQYBT, MD, and sEMG on the pain side (PS) and no pain side (NPS) of PG

Variable	PS	95% CI	NPS	95% CI	*p*
*UQYBT (%LL)*					
Medial	118.4 ± 21.3	109.2 to 127.6	117.9 ± 19.1	109.6 to 126.2	0.978
Inferolateral	74.6 ± 14.9	68.1 to 81.1	74.9 ± 14.1	68.8 to 81.1	0.691
Superolateral	55.4 ± 14.7	49.0 to 61.8	57.0 ± 14.3	50.8 to 63.2	0.530
Composite	82.8 ± 14.2	76.7 to 89.0	83.4 ± 14.2	77.2 to 89.5	0.704
*MD (N)*					
ER	122.2 ± 38.6	105.5 to 138.9	126.2 ± 39.9	108.9 to 143.5	0.853
IR	113.1 ± 38.5	96.5 to 129.8	116.2 ± 36.4	100.5 to 132.0	0.684
ABDU	84.8 ± 26.8	73.1 to 96.4	90.8 ± 25.5	79.8 to 101.9	0.455
ADDU	95.0 ± 35.0	79.8 to 110.1	92.5 ± 29.3	79.8 to 105.2	0.746
*sEMG (%MVC)*					
UP	50.4 ± 11.2	45.5 to 55.2	53.5 ± 12.1	48.3 to 58.8	0.363
LT	47.7 ± 7.2	44.5 to 50.8	49.9 ± 8.3	46.2 to 53.5	0.346
PM	53.4 ± 12.7	47.9 to 58.9	53.3 ± 14.7	46.9 to 59.7	0.983

The values were presented as mean and standard deviation (% LL: percentage of limb length; N: Newton; % MVC: percentage of maximum voluntary contraction)

### Intergroup comparison

Comparison of the UQYBT, MD, and sEMG between PG and the NPG was performed using data from the side with pain and the dominant side, respectively. There was a statistically significant difference in sEMG for muscle activity in the lower trapezius (*p* = 0.038), with Cohen's *d* = 0.51 (medium effect size). The other variables showed no significant differences (Table 4).

**Table 4:** Comparison of the variables UQYBT, MD, and sEMG on the dominant side (DS) and non-dominant side (NDS) of NPG

Variable	DS	95% CI	NDS	95% CI	*p*
*UQYBT (%LL)*					
Medial	110.3 ± 10.5	107.2 to 113.4	112.0 ± 10.6	108.9 to 115.2	0.410
Inferolateral	72.1 ± 17.4	67.0 to 77.2	73.7 ± 15.3	69.2 to 78.2	0.652
Superolateral	53.0 ± 10.2	50.0 to 56.0	55.5 ± 10.8	52.4 to 58.7	0.239
Composite	78.4 ± 10.2	75.4 to 81.4	80.5 ± 9.7	77.6 to 83.4	0.336
*MD (N)*					
ER	134.8 ± 35.7	124.3 to 145.3	125.8 ± 37.2	114.9 to 136.8	0.221
IR	123.1 ± 40.6	111.1 to 135.0	119.6 ± 37.2	108.6 to 130.5	0.583
ABDU	100.3 ± 29.8	91.6 to 109.1	97.3 ± 29.9	88.5 to 106.1	0.632
ADDU	108.7 ± 43.7	95.8 to 121.6	102.4 ± 38.6	91.1 to 113.7	0.433
*sEMG (%MVC)*					
UP	52.1 ± 8.5	49.6 to 54.6	50.9 ± 8.1	48.5 to 53.3	0.490
LT	51.9 ± 8.9	49.3 to 54.6	52.7 ± 9.6	49.8 to 55.5	0.694
PM	58.6 ± 10.9	55.4 to 61.9	57.0 ± 10.6	53.9 to 60.1	0.458

The values were presented as mean and standard deviation (% LL: percentage of limb length; N: Newton; % MVC: percentage of maximum voluntary contraction)

### Association between training pain reports and training variables and ADLs

There was evidence of a statistically significant positive association between the categorical variable DASH and pain. The other variables showed no evidence of a statistically significant associating with shoulder pain. Table 5 presents the results of Chi-square test for categorical or ordinal variables values.

**Table 5:** Comparison of the variables UQYBT, MD, and sEMG on the PG and NPG

Variable	PG	95%CI	NPG	95%CI	*p*
*UQYBT (%LL)*					
Medial	118.4 ± 21.3	109.2 to 127.6	110.3 ± 10.5	107.2 to 113.4	0.055
Inferolateral	74.6 ± 14.9	68.1 to 81.1	72.1 ± 17.4	67.0 to 77.2	0.677
Superolateral	55.4 ± 14.7	49.0 to 61.8	53.0 ± 10.2	50.0 to 56.0	0.625
Composite	82.8 ± 14.2	76.7 to 89.0	78.4 ± 10.2	75.4 to 81.4	0.289
*MD (N)*					
ER	122.2 ± 38.6	105.5 to 138.9	134.8 ± 35.7	124.3 to 145.3	0.854
IR	113.1 ± 38.5	96.5 to 129.8	123.1 ± 40.6	111.1 to 135.0	0.984
ABDU	84.8 ± 26.8	73.1 to 96.4	100.3 ± 29.8	91.6 to 109.1	0.197
ADDU	95.0 ± 35.0	79.8; 110.1	108.7 ± 43.7	95.8; 121.6	0.851
*sEMG (%MVC)*					
UP	50.4 ± 11.2	45.5; 55.2	52.1 ± 8.5	49.6; 54.6	0.521
LT	47.7 ± 7.2	44.5; 50.8	51.9 ± 8.9	49.3; 54.6	**0.038**
PM	53.4 ± 12.7	47.9; 58.9	58.6 ± 10.9	55.4; 61.9	0.098

No significant difference were found (*p* > 0.1)

The values were presented as mean and standard deviation (% LL: percentage of limb length; N: Newton; % MVC: percentage of maximum voluntary contraction)

### Association with muscle strength

Tables 6 show the values of the association analysis by simple linear regression of the variable, namely, functional tests (dependent) and muscle forces (independent). In the NPG, there was evidence of a statistically significant positive association between the UQYBT (medial component) and CKCUEST tests with the evaluated muscle forces (*p* < 0.05) and the abductor strength for the superolateral component of the UQYBT.

**Table 6:** Sample values and percentage of the various variables (age, sex, practice time, weekly hours, and DASH values) categorized, and the *p* value in association with the sub-items (without pain and with pain)

Variable	Categorical variables	No pain	Pain	*p* value
Age (years)	< 20	3 (3.8%)	0	0.42
	20–29	21 (26.6%)	12 (15.2%)	
	30–39	22 (27.8%)	16 (20.3%)	
	> 40	4 (5.1%)	1 (1.3%)	
Sex	M	32 (40.5%)	17 (21.5%)	0.63
	F	18 (22.8%)	12 (15.2%)	
Practice time	< 1 year	7 (8.9%)	6 (7.6%)	0.45
	1–2 years	16 (20.3%)	9 (11.4%)	
	2–3 years	12 (15.2%)	3 (3.8%)	
	> 3 years	15 (19.0%)	11 (13.9%)	
Week hours	2–5 h	25 (31.6%)	12 (15.2%)	0.27
	6–10 h	17 (21.5%)	15 (19.0%)	
	11–18 h	8 (10.1%)	2 (2.5%)	
DASH	0–20	49 (62.0%)	22 (27.8%)	**0.004**
	21–40	1 (1.3%)	6 (7.6%)	
	41–60	0	1 (1.3%)	
	61–80	0	0	
	81–100	0	0	
DASH (OPC 1)	0–20	41 (51.9%)	15 (19.0%)	**0.03**
	21–40	5 (6.3%)	9 (11.4%)	
	41–60	3 (3.8%)	3 (3.8%)	
	61–80	1 (1.3%)	2 (2.5%)	
	81–100	0	0	
DASH (OPC 2)	0–20	46 (58.2%)	21 (26.6%)	**0.05**
	21–40	3 (3.8%)	5 (6.3%)	
	41–60	1 (1.3%)	3 (3.8%)	
	61–80	0	0	
	81–100	0	0	

No significant difference were found (*p* > 0.1)

For the PG, this pattern was not observed, only evidencing a statistically significant positive association between the strength of external shoulder rotators and the medial component of the UQYBT (*p* < 0.05). The other strength variables showed no evidence of a statistically significant association with the tests.

## Discussion

Most of the published scientific literature on Crossfit shows that the shoulder is the most frequently injured joint. However, few studies have verified whether there is a difference in the biomechanical and functional characteristics between Crossfit athlete with and without shoulder pain [14, 17, 43–47].

The main findings of this study were that the lower trapezius in Crossfit athletes with shoulder pain shows lower activation at sEMG compared to Crossfit athletes without shoulder pain, with no difference in functional tests. Regarding the DASH questionnaire, the highest score was associated with the presence of shoulder pain, both in daily activities and in sports. In addition, demographic factors, such as age, height, and weight, do not differ between participants with and these without shoulder pain, with no difference in practice time and weekly training hours.

The Crossfit athletes with and without shoulder pain were otherwise comparable, with no relationship between demographic and anthropometric data and the presence of shoulder pain [14, 16, 47].

Regarding the training characteristics, our study showed that participants with and without shoulder pain did not differ in terms of total practice time and hours of weekly training, contrary to what highlighted by other investigations [14, 16, 43, 46]. However, training volume and the competitive level are related to the risk of injury given the exposure to joint overload [14, 46].

Previous studies in different populations, regarding age, activity level and sports modality, have shown a definite relationship between pain and function of the upper limb, suggesting that the DASH questionnaire allows to assess the loss of functional capacity in symptomatic patients. Therefore, subjects with pain may exhibit impaired performance in functional tests of the upper limb, such as CKCUEST and UQYBT. However, this was not verified in the present study: the Crossfit athletes involved in the present investigation showed similar performance in CKCUEST and UQYBT regardless of the reported pain in their shoulder. In practice, although some athletes had a lower functional capacity (implied by the higher DASH score) and reported pain during training, they had no difficulty in performing the various functional tests.

Indeed, there are contradicting reports on the relationship between CKCUEST performance and shoulder symptoms. Tucci et al. [22] found a significant difference when comparing the CKCUEST score of individuals at different levels of physical activity and symptoms. Individuals with shoulder injuries were able to perform the lowest average number of touches on the test, while healthy assets had the highest scores. In another study, Pontillo, Spinelli, & Sennett [48] observed that those who scored lower on CKCUEST were more likely to suffer from a shoulder injury.

On the other hand, subjects with and without shoulder symptoms did not differ in CKCUEST score, in agreement with the result of the present investigation [11, 21]. Such contradictions can be explained by the fact that the CKCUEST is executed in a position that demands from the individual not only shoulder, but also trunk stability, in addition to strength and speed. In addition, it is not possible to analyse the performance of the limbs separately, considering that it is a bilateral test. The results, therefore, can be influenced by factors other than the stability of the shoulder complex.

In the present study, the performance on the UQYBT test did not differ between PG and NPG. However, the few studies which evaluated the results of this test compared one side versus the other and did not compare participants with and without shoulder symptoms. To our knowledge, only one study assessed the outcome of UQYBT in participants with and without shoulder impingement syndrome. There was a significant difference in the participants' performance in the medial and inferolateral directions, patients with shoulder injuries perform worse in the UQYBT than healthy subjects [49]. However, the authors examined a smaller population and non-athletic participants, making it difficult to compare the results with the present study. Therefore, future studies which evaluate appropriate populations are necessary to compare the results.

Most authors agree that there is no significant difference between the dominant and non-dominant side in the performance of UQYBT [18, 19, 23, 31, 50]. The present study also showed no significant difference in this test between the dominant and non-dominant side of the NPG and between the painful side and the pain free side of the PG. The reach in the medial direction was greater, followed by the inferolateral and superolateral direction, as in other studies, and the non-dominant side performed better in all directions [23, 51].

This study also verified a lower activation of the lower trapezius muscle in the subjects with shoulder pain during a lifting movement of the bar (shoulder press), compared with the participants without shoulder pain. The activity of the other muscles evaluated (upper trapezius and pectoralis major) did not show any difference, and the isometric strength of the rotator cuff muscles was equally not different. Scapulothoracic muscle strength and activity in symptomatic patients has been widely investigated, although not in Crossfit practitioners. Aerial movement sports seem to influence scapulothoracic muscle balance. Symptomatic athletes present a reduction in the lower trapezius activity and an increase in the upper trapezius activity. and the increase in load tends to increase the intensity of the muscular activity [24, 42, 52]. Athletes whose sport involves aerial movements showed higher muscle strength compared to athletes from other sports [53]. In this case, regardless of the sport, the upper trapezius was stronger than any other muscle evaluated, and the lower trapezius was the one with the lowest value in all groups.

Regarding isometric strength, the present study identified no significant difference between participants with and without shoulder pain. This result contradicts other studies, which found that shoulder symptoms can affect the strength of external rotators, internal rotators and abductors [6, 54, 55].

We also demonstrated a different pattern of muscle forces associated with functional tests regardless of whether our study participants reported pain or not during training. Individuals with shoulder pain have significantly lower isometric strength of the rotator cuff muscles on the injured side, and this may be associated with the diagnosis of injury ^54,55^.

Crossfit combines movements from various sports and high loads in weightlifting. Therefore, Crossfit practitioners, as well as athletes in other sports which require shoulder movements above 90° at high load and speed, can adjust and compensate to stabilize the scapula during exercises and thus, achieve their training objectives. This could explain the fact that there are no significant differences in muscle activity and isometric strength between the groups evaluated, except in the activity of the lower trapezius. In addition, in the electromyographic assessment of muscle activity, the shoulder press was performed with a 15 kg bar by all participants regardless of their body mass and performance capabilities. This relatively low load may not have reproduced the muscle activity that occurs during high load weightlifting exercises, which may also have influenced the fact that no significant difference was found in the activation of the other muscles.

This study had limitations. The inclusion in the painful shoulder group was based on self-report, and a clinical and/or imaging diagnosis was not performed. The electromyographic muscle activity test was not performed at full load and may not have simulated the actual activation of the muscles during actual training. Further studies on Crossfit should better address the biomechanical feature of the sport, and study their relationship with symptoms.

## Conclusion

Crossfit practitioners who report shoulder pain during training showed less activation of the lower trapezius, but good mobility and function of the shoulder complex. Scapular stabilization work, with emphasis on the activation of the lower trapezius, is suggested as a priority for Crossfit athletes. In addition, the quality of the execution of movements with loads above 90° is important. As these are the most aggressive exercises on the shoulder, it is necessary that the stabilizing muscles of the scapula are activated in a balanced fashion, preventing injuries to the shoulder joint.

## Data Availability

The datasets generated and/or analysed during the current study are not publicly available due data that are the private property of the authors prior to publication of the study may be compromised but are available from the corresponding author on reasonable request.

## Abbreviations

CONSORT: Consolidated Standards of Reporting Trials
VAS: Visual analogue pain scale
DASH: Disability Arm Shoulder and Hand questionnaire
UQYBT: Upper Quarter Y Balance Test
LL: Limb length
CKCUEST: Closed Kinetic Chain Upper Extremity Stability
ER: External rotation
IR: Internal rotation
ABDU: Abduction
ADDU: Adduction
N: Newton
sEMG: Surface electromyography
UT: The upper trapezius
LT: Lower trapezius
PM: Pectoralis major
MVC: Maximum voluntary contraction
